# Glaucoma Symptom Scale: Psychometric properties of the Serbian version

**DOI:** 10.1371/journal.pone.0216920

**Published:** 2019-05-20

**Authors:** Ivan Sencanic, Tatjana Gazibara, Jelena Dotlic, Miroslav Stamenkovic, Vesna Jaksic, Marija Bozic, Anita Grgurevic

**Affiliations:** 1 Clinic for Eye Disease “Prof. dr Ivan Stankovic”, University Medical Center “Zvezdara”, Belgrade, Serbia; 2 Institute of Epidemiology, Faculty of Medicine, University of Belgrade, Belgrade, Serbia; 3 Faculty of Medicine, University of Belgrade, Belgrade, Serbia; 4 Clinic of Obstetrics and Gynecology Clinical Center of Serbia, Belgrade, Serbia; 5 Faculty of Special Education and rehabilitation, University of Belgrade, Belgrade, Serbia; 6 Clinic for Eye Disease “Prof. dr Djordje Nesic”, Clinical Center of Serbia, Belgrade, Serbia; Flinders University Faculty of Medicine Nursing and Health Sciences, AUSTRALIA

## Abstract

**Purpose:**

The study aim was to translate and validate the Glaucoma Symptom Scale (GSS) in Serbian language.

**Methods:**

Clinical parameters and socio-demographic data were collected for each of the 177 enrolled glaucoma patients. Each eye was classified according to the Glaucoma staging system by Mills into 6 stages. Patients filled out the GSS and National Eye Institute Visual Function Questionnaire (NEI-VFQ 25). The GSS comprises 10 complaints common for glaucoma patients on a topical treatment, grouped into two subscales: SYMP-6 (non-visual) and FUNC-4 (visual problems). The GSS was translated following the customary methodology and its psychometric properties were assessed by using both Classical Test Theory (CTT) and Rasch analysis.

**Results:**

The internal consistency of the Serbian GSS for the whole scale was very good (Cronbach’s alpha = 0.81). On factor analysis items were clustered into 2 factors (48.92% of variance) which corresponded to the original scale. The total and subscale GSS scores correlated significantly with measures of disease severity and also with total score and analogous NEI-VFQ 25 subscale scores. In Rasch analysis we obtained adequate item reliability index (0.90). Almost all items had infit and outfit mean squares in the accepted range. However, measurement precision was poor (low person separation reliability) and targeting revealed a ceiling effect.

**Conclusion:**

When analyzed with CTT the Serbian version of the GSS seems to be a valid instrument, but Rasch analysis revealed some serious measurement flaws, therefore it should not be used in its current format. Further studies to modify and improve GSS are needed prior to its application for Serbian glaucoma patients.

## Introduction

Glaucoma is a chronic neurodegenerative disease which, if left untreated, can lead to permanent visual field deficits that can ultimately result in blindness [1, 2]. While some of glaucoma patients have no problems in everyday functioning, others, especially with an end-stage disease, report problems in performing daily activities and objective clinical measurements cannot adequately describe all of their limitations [3, 4]. The standard medical treatment of glaucoma often involves use of topical glaucoma medications. This therapy may produce side effects (due to active drug or preservatives) and induce ocular surface disease (OSD), which may further deteriorate health status and functioning [5]. Thus, visual disability scales are commonly used to evaluate change in patients day-to-day functioning and health-related quality of life (HRQoL) [6–8].

Two methods that are generally used for developing and validating HRQoL instruments are classical test theory (CTT, including calculation of Cronbach’s alpha coefficient, exploratory and confirmatory factor analysis, correlations) and item response theory (IRT) with Rasch analysis being the most commonly used IRT model. Limitations of the CTT have previously been acknowledged and are mostly related to the inability to distinguish item difficulty level as well as to categorize orders of item responses [9, 10]. This is remedied by Rasch analysis, a modern and more sophisticated method that transforms raw category level item responses into interval-level scale. It provides a more reliable insight in difficulty level of items in relation to one’s relative abilities. Interval-level scores improve measurement precision and its sensitivity [11–16]. Also, Rasch models offers a more comprehensive assessment of psychometric features of a questionnaire such as unidimensionality, targeting, measurement precision, response category functioning [14–16]. For this reason, the Rasch method of assessing instruments has been increasingly used in ophthalmologic research.

The Glaucoma Symptom Scale (GSS) is a simple and brief glaucoma-specific questionnaire aimed at quantifying complaints and functional impairment in patients with glaucoma [17]. The GSS focuses on two most important aspects of glaucoma: one is the functional glaucoma-related disability and the other is treatment-related symptomatology. The English version was developed using the CTT and, thus far, has been translated and validated in Italian and Spanish populations. These three versions of the GSS underwent an assessment of psychometric properties using the CTT and the results have showed adequate validity and reliability [11–19]. However, when Rasch analysis of the English version was performed in Australian [20] and Indian [21] glaucoma populations, poor psychometric properties were reported. Despite the observed drawbacks, the GSS questionnaire is still being used in recent studies in its original form [22–24].

Factors associated with HRQoL in Serbian glaucoma population have not been previously examined, mainly because of the lack of appropriate instruments for measurement of glaucoma-related impact on one’s health status. Therefore, translation of the first original version of the GSS to Serbian language would provide important information related not only to HRQoL of glaucoma patients in this population, but also regarding its advantages and disadvantages.

The primary aim of our study was to translate the Glaucoma Symptom Scale to Serbian language and to assess its psychometric properties. Our goal was also to determine whether the characteristic of the GSS developed in one population differ when it is used in another sociodemographic setting, as it has previously been shown that the use of one questionnaire can be dependent on numerous factors such as culture, language, social context etc. [25–28]. Secondly, we aimed at comparing the results of psychometric evaluations using traditional CTT and modern Rasch model, since previous findings in literature suggest inconsistencies between these two analytic methods. As a result, this study is the first outside of the English-speaking setting to evaluate the GSS using Rasch analytic approach.

## Methods

### Participants

Study participants were glaucoma patients who came for regular check-up between August 2015 and September 2016 at the Clinic for Eye Disease University Medical Center “Zvezdara”.

The inclusion criteria for the study were: age 18 years and older with glaucoma diagnosed at least one year prior to recruitment. Diagnosis of glaucoma was made based on the presence of glaucomatous optic discneuropathy (neuroretinal rim thinning with a vertical cup to disc ratio of equal or more than 0.6, or inter-eye asymmetry of optic disc cupping of more than 0.2 and/or presence of focal notching characteristic of glaucoma) associated with visual field (VF) defects in either eye [29]. A glaucomatous visual field loss was defined if the following were present on OCTOPUS standard automatic perimetry: 1) mean defect greater than 2 db; 2) loss of variance greater than 6 db; 3) at least 7 points with sensitivity decreased of more than 5 db and at least 3 contiguous points) [30]. Patients with primary open angle glaucoma (POAG), primary angle closure glaucoma (PAGC), pseudo-exfoliative (PEX), normal tension glaucoma (NTG) and pigmentary glaucoma (PG) were included in this study. Patients with POAG had open angle on gonioscopy and intraocular pressure (IOP) of 21mmHg and higher, while NTG patients were accompanied by a normal IOP. Diagnosis of PACG was established by the presence of elevated IOP and a closed iridocorneal angle on gonioscopy (posterior trabecular meshwork not visible in more than 180 degrees of the angle, and/or peripheral anterior synechiae- pathologic adhesions of the iris at the level of the anterior trabecular meshwork or higher). The PEX was characterized by the exfoliation material on the pupil border and on the surface of the anterior lens capsule and PG was defined by a pigment dispersion syndrome [31].

The exclusion criteria were: severe vision-impaired eye diseases such as: significant corneal opacities, clinically significant cataract (grade 2 or more by Lens Opacities Classification System III) [32] and age-related macular degeneration. Patients with high refractive errors (> 5 dioptres sphere and/or cylindrical errors > 2 dioptres cylinder), patients with history of intraocular surgery in the previous 3 months were also excluded from the study. Additionally, patients were excluded if they presented any of the following: major neurological, psychiatric, cognitive or orthopedic disturbances that could affect their vision and HRQoL.

Each patient had a complete ophthalmological examination, including a comprehensive clinical assessment of glaucoma. For each eye we also collected information on number of glaucoma medications per day, number of drops per day, history and number of previous trabeculectomy surgery. In order to correlate glaucoma-specific HRQoL as measured by the GSS with clinical indices, we measured visual acuity (VA) and visual field (VF). Snellen VA was recorded and converted to the logarithm of the Minimal Angle of Resolution (logMAR) for data analysis. A central 30 degrees of the visual field was tested using automated static perimetry (OCTOPUS 600, Haag—Streit Eye Suite). Patients with unreliable VF (e.g. fixation loss, reliability factor exceeding 15%) were not included in the study. The VF mean defect (MD) and square root of loss variance (sLV) were also registered. Based on the visual fields’ MD results, the eye with better overall sensitivity was registered as the better eye and the contralateral eye as the worse eye. Subsequently, each eye was categorized into one of the following glaucoma stage respecting the Glaucoma staging system. This system was introduced by Mills and associates and it was designed based on a thorough literature review of previously described glaucoma staging systems [33]. It represents a comprehensive and accurate method for glaucoma staging on the basis of the visual field. The following stratification to 5 different stages is based on the Octopus visual field results: stage 0 (earliest glaucoma, MD≤-0.8db), stage 1 (early glaucoma, from -0.7dbto +4.4 db), stage 2 (moderate glaucoma, MD form +4.5db to +9.5db), stage 3 (advanced glaucoma, MD form +9.6db to +15.3db), stage 4 (severe glaucoma, MD from +15.4db to 23.1db) and stage 5 (end-stage glaucoma, MD≥23.2db).

This research was approved by the Ethics Committee of the Faculty of Medicine, University of Belgrade and signed informed consent was obtained from all participants before enrolment.

### Glaucoma Symptom Scale

The Glaucoma Symptom Scale was designed in the late nineties as a modified version of Ocular Hypertension Study checklist that was used to evaluate glaucoma medication’s side effects. The GSS comprise 10 complaints (6 of which are non-visual, 4 of which are visual) that are commonly reported by glaucoma patients on a topical treatment. These items are grouped into two subscales: SYMP-6, characterized by non-visual problems and FUNC-4 characterized by visual problems. Initially, patients were asked whether or not they had specific symptom for each eye separately within the past 4 weeks. If so, they were asked to mark on a 5-level scale how troublesome was the symptom (0 for a very troublesome and 4 if the complaint was absent). Later on, this score was converted to a 0 to 100 scale, with 0 showing presence of a prominent problem and 100 representing absence of complaints. Total score is the mean of the sum of all 10 scores averaged between the two eyes. Scores can be derived for each eye individually. Subscale scores are mean of the sum of the item-level subscale scores averaged between the 2 eyes. Lower total score and subscale scores are indicating poorer HRQoL [17].

### Translation of the Glaucoma Symptom Scale

The Serbian version of the GSS was developed following the customary methodology for translation of a questionnaire [34]. The goal of this procedure is to design a version that is semantically and conceptually analogous to the original instrument. Translation of the GSS to Serbian language was completed by two independent translators i.e. “forward translation”. Then, a third translator not working in medical field, who was blinded to the original questionnaire, performed “back translation” (from Serbian back to English). Afterwards, the back-translated GSS was compared with the original English version and some slight discrepancies in the translations were reconciled. In order to assess clarity, simplicity and comprehension of the translated items by the Serbian glaucoma population, GSS was pre-tested on 15 glaucoma patients.

No remarks related to understanding and clarity of items were registered, thus the final version was established and applied in this study. The GSS was completed by the patients in the presence of physician.

### National Eye Institute Visual Function Questionnaire (NEI-VFQ 25)

The NEI-VFQ 25 questionnaire is a vision-specific quality of life instrument, most widely used in vision-targeted HRQoL assessments [35]. It has 25 items organized into 12 subscales: (general health, general vision, ocular pain, difficulty with near-vision activities, distance-vision activities, limitation of social functioning because of vision, mental health problems because of vision, role limitations because of vision, dependency on others because of vision, driving difficulties, difficulty with color vision and difficulty with peripheral vision). The total score is the mean of all items except for general health. The NEI-VFQ 25 has been previously translated to Serbian language and it was shown to be valid and reliable instrument for the assessment of vision specific HRQoL in Serbian population [36]. In the present study, NEI-VFQ 25 was used to correlate vision specific HRQoL questionnaire with glaucoma-specific HRQoL scale.

### Data analysis

To depict the GSS scale, we evaluated minimum and maximum scores for each item and for both GSS subscales. Clinical data of glaucoma patients were analyzed using SPSS statistical software (Version 21.0 SPSS Science).

To check stability (i.e. test-retest) of the questionnaire, 133 of 177 glaucoma patients repeated the GSS testing after two week period had expired. Stability was evaluated using the Spearman’s correlation coefficient.

### Internal consistency

Internal consistency of the GSS and both subscales was assessed using Cronbach’s alpha coefficient. This coefficient shows correlations between scale items and is the classic estimate of the reliability of a psychometric test. Cronbach’s alpha is a function of the scale items number, the average covariance between item-pairs, and the variance of the total score. Values above 0.7 are considered statistically adequate [37]. We used multivariate Hotelling T–square test (HT^2^) that implies that all items on a scale have the equivalent mean. This test evaluates significance of differences between acquired mean score values of all GSS items together and the hypothetic case in which items have identical scores.

Corrected Item–Total Correlation (CI–TC) analysis was performed to assess discriminating characteristics of the scale items. This analysis can show if an item is inconsistent with the averaged results of other items. It examines the relationships of one item with the score of remaining scale items. Based on the significance of these correlations the CI-TC analysis demonstrates whether or not the item should belong to a scale. Appropriate values of CI–TC for an item are ≥ 0.40 [37].

### Construct validity

Construct validity evaluates the degree to which an instrument/test measures the intended construct. Evaluation of construct validity requires examination of questionnaire/scale items correlations with variables that are known to be related to the construct based on previous data and test. In order to asses construct validity, an exploratory factor analysis–EFA with Varimax rotation was performed [37]. This analysis identifies complex interrelationships among scale items and it groups the items based on strong correlations providing a factor structure in the scale. Significant factors i.e. domain within the scale, have eigenvalue higher than 1.0. Eigenvalues measure the amount of variation in the total sample accounted for by each factor. Observed factors should be comparable with the originally established for the examined questionnaire/scale. Factor loadings on the EFA represent correlation coefficients between the scale items and established factors. The communality index defines the variance of the scale item accounted for all factors. It represents the sum of squared factor loadings (i.e. percent of variance) for all factors included in the given scale item. Higher communalities are better, while index <0.4 indicates items that should be removed from the scale [37].

### Criterion validity

Criterion validity is the extent to which a measure is related to an outcome. It is usually assessed through comparison with well-established measurement that acts as the criterion against which the new instrument is assessed. In our study criterion validity of the GSS was assessed by exploring the mean scale score and mean subscales scores with patients' clinical characteristics (VA, MD, sLV, glaucoma stage, number of glaucoma medication/drops per day, number of trabeculectomies) and with NEI-VFQ 25 total and subscale scores using Spearman’s correlation coefficient rho (ρ) [37].

### Psychometric properties in Rasch analysis

Rasch analysis is a mathematical probability-based model frequently used in testing of psychometric characteristics of questionnaires [13–16, 25, 28, 38]. It evaluates person’s ability compared to an item difficulty and the results are calculated in log-odds units (logits). Item difficulty and person ability are shown on an interval scale (chart) with logits representing measurement units. Positive (upper) part of the scale illustrates items with greater difficulty and persons with higher ability, while negative (lower) side shows persons with lower ability and less difficulty items [13–16,25,28,38]. Winsteps software (version 4.0.1) was used to perform the Rasch analysis.

According to Rasch models, basic elements that describe instrument properties are dimensionality, measurement precision and targeting.

Unidimensionality refers to questionnaire’s ability to measure one single construct. It is an indicator of good validity and it is described by fit statistics assessment (infit and outfit) and principal component analysis (PCA) of the residuals [14–16, 39, 40]. Both infit and outfit statistics define how well each item matches the underlying construct (infit statistics is better indicator because it is less sensitive to effect of the outliers). Mean square standardized residuals (MNSQ) are being used to characterize fit statistics and the ideal value of MNSQ is 1.0. Preferred values range from 0.7 to 1.3, but less strict criteria consider an interval between 0.5 and 1.5 to be adequate as well [41,42]. In the PCA, a good indicator of unidimensionality would be if at least 60% of raw variance is explained by the questionnaire. Unidimensionality is also confirmed if the eigenvalue of the unexplained variance of the first contrast is less than 2.0 [15,16,39,40].

Person separation reliability (PSR) or index (PSI) illustrates measurement precision of an instrument and it clarifies how many levels of person ability a questionnaire can differentiate. A PSR value of 0.8 (PSI≥2.0) suggests that three groups of disabilities are being identified and it is a minimal accepted value of discrimination. Item separation denounces the reproducibility of the instrument and the number of item groups according to their difficulty. If separation indices are ≥0.8 the scale/questionnaire is well constructed [38]

Targeting is assessed by evaluating person-item interval scale that illustrates how the person ability matches the level of item difficulty. Perfectly targeted instrument has a scale with both person and item mean located at the same level of 0 logit. Nevertheless, if mean person and mean item differ up to 1 logit targeting is still adequate. Mistargeting is referred when the mean difference is over 1 logit [15, 16, 39, 40]. Person-item interval scale can also reveal how the items are being grouped at a particular section along the scale and poor targeting is present if there is ceiling and/or floor effect (clustering of the responses at the very ends of the scale scores) [43].

## Results

### Description of the study sample

Study included 177 patients (113 women and 64 men) with mean ± SD age of 62.8 ± 13.6 years. Stratification by disease severity showed that in the case of better eye almost half of the eyes had minimal or early VF defects (stage 0 in 12.2% eyes and stage 1 in29.7% eyes). In the case of worse eye, stage 1 was noted in 24.2%, while advanced, severe and end-stage VF defects were registered in 7.6%, 3.7% and 7.3% of eyes respectively. Most of the eyes had no previous trabeculectomy (89.3% of better eyes and 79.7% of worse eyes). Types of glaucoma, number of glaucoma medications/drops per day per eye and other clinical characteristics of patients are presented in Table 1.

**Table 1:** Demographic and clinical characteristics of Serbian patients with glaucoma.

Patients' characteristics		Overall sample	
Mean age ± SD (years)		62.83 ± 13.60	
Sex, n (%)	female	113 (63.8)	
	male	64 (36.2)	
Type of glaucoma, n (%)	POAG	80 (45.2)	
	PACG	28 (15.8)	
	PEX	28 (15.8)	
	PG	15 (8.5)	
	NTG	26 (14.7)	
Patients' characteristics		Better eye	Worse eye
Visual acuity (logMAR) mean + SD		0.08 ± 0.11	0.68 ± 1.58
Visual field (db) mean + SD	MD	2.38 ± 5.04	7.65 ± 9.37
	sLV	4.01 ± 3.05	4.71 ± 3.08
Glaucoma stage, n (%)	0, no or minimal defects	40 (12.2)	19 (5.8)
	1, early defects	97 (29.7)	79 (24.2)
	2, moderate defects	22 (6.7)	18 (5.5)
	3, advanced defects	11 (3.4)	25 (7.6)
	4, severe defects	7 (2.1)	12 (3.7)
	5, end-stage disease	0 (0)	24 (7.3)
Number of trabeculactomies, n (%)	none	158 (89.3)	141 (79.7)
	one	19 (10.7)	29 (16.4)
	two	0 (0)	7 (4.0)
Number of glaucoma medications per day, n (%)	none	6 (3.4)	11 (6.2)
	one	40 (22.6)	36 (20.3)
	two	50 (28.2)	58 (32.8)
	three	66 (37.3)	59 (33.3)
	four	15 (8.5)	13 (7.3)
Number of drops per day, n (%)	none	6 (3.4)	12 (6.9)
	one	47 (27.0)	45 (25.9)
	two	11 (6.3)	13 (7.5)
	three	76 (43.7)	72 (41.4)
	four or more	34 (23.0)	32 (14.2)

GL—glaucoma; SD—standard deviation; POAG—primary open angle glaucoma; PACG—primary angle closure glaucoma; PEX—pseudo-exfoliative; PG—pigmentary glaucoma; NTG—normal tension glaucoma; logMAR—logarithm of the Minimal Angle of Resolution; MD—mean defect; sLV—square root of loss

### Glaucoma symptom scale scores

We have not observed any major changes in the translation process from English to Serbian and back to English language.

The mean total score for the Serbian GSS was 87.52 ± 14.04 for both eyes. This score suggests that the level of HRQoL among our patients with glaucoma was favorable. Subscale scores were lower in SYPM-6 than in FUNC-4 subscales and they measured 85.08 ± 17.93 and 91.19 ± 12.65 respectively. The minimum and maximum values of items subscale and total scores of the Serbian GSS for better, worse eye and for both eyes are shown in Table 2.

**Table 2:** Average scores on Glaucoma Symptom Scale in Serbian language according to items and subscales.

GSS items	Better eye (n = 177)			Worse eye (n = 177)			Both eyes (n = 177)		
	min	max	mean, SD	min	max	mean, SD	min	max	mean, SD
1. Burning, Smarting, Stinging	0	100	85.31 (26.97)	0	100	84.32 (27.40)	0	100	84.99 (25.11)
2. Tearing	0	100	87.99 (23.39)	0	100	87.43 (24.00)	0	100	87.70 (23.21)
3. Dryness	0	100	76.69 (29.98)	0	100	76.13 (30.01)	0	100	76.41 (29.67)
4. Itching	0	100	84.18 (25.36)	0	100	83.76 (25.44)	0	100	83.97 (24.92)
5. Soreness, Tiredness	0	100	87.57 (24.30)	0	100	86.86 (25.57)	0	100	87.21 (24.46)
6. Blurry/Dim vision	0	100	88.84 (22.75)	25	100	87.01 (24.00)	25	100	87.64 (22.17)
7. Feeling of something in your eye	0	100	90.11 (23.26)	0	100	89.83 (24.03)	0	100	90.19 (22.21)
8. Hard to see in daylight	25	100	94.77 (16.98)	25	100	94.63 (17.46)	25	100	94.71 (16.67)
9. Hard to see in dark places	25	100	87.01 (23.85)	25	100	86.72 (23.69)	25	100	86.86 (23.32)
10. Halos around lights	0	100	95.48 (15.56)	0	100	95.62 (15.49)	0	100	95.55 (15.50)
**GSS subscales**									
SYMP-6	0.00	100.00	85.57 (17.81)	0.00	100.00	84.79 (18.88)	0.00	100.00	85.08 (17.93)
FUNC-4	31.25	100.00	91.49 (12.65)	31.25	100.00	90.83 (13.45)	31.25	100.00	91.19 (12.65)
**Total GSS score**	30.00	100.00	87.94 (13.93)	30.00	100.00	87.29 (14.95)	30.00	100.00	87.52 (14.04)

GSS—Glaucoma Symptom Scale; SD—standard deviation; p—probability value

Stability of the Serbian version of the GSS as measured by the Spearman’s correlation coefficient was very good (ρ = 0.90, p<0.001).

### Internal consistency

Cronbach’s alpha coefficient for the whole scale measured 0.81 for both eyes. Internal consistency was also very good for SYMP-6 subscale (Cronbach’s alpha 0.80). Nevertheless, FUNC-4 subscale had somewhat lower coefficient and Cronbach’s alpha was 0.53 for both eyes (Table 3).

**Table 3:** Cronbach’s alpha coefficients, interclass correlation coefficients and corrected item-total correlation coefficients for the Serbian version of the Glaucoma Symptoms Scale for both eyes.

GSS–items and subscale scores	Cronbach’s alpha	Cronbach’s alpha if item deleted	CI—TC
**SYMP-6**	0.81		
1. Burning, Smarting, Stinging		0.77	0.65
2. Tearing		0.78	0.58
3. Dryness		0.78	0.57
4. Itching		0.80	0.47
5. Soreness, Tiredness		0.79	0.51
7. Feeling of Something in Your Eye		0.78	0.61
**FUNC-4**	0.53		
6. Blurry/Dim vision		0.80	0.46
8. Hard to See in Daylight		0.81	0.29
9. Hard to See in Dark Places		0.81	0.33
10. Halos around lights		0.81	0.38
**Total GSS score**	0.81		

GSS—Glaucoma Symptom Scale; CI–TC—Corrected Item–Total Correlation coefficient.

The values of the CI-TC coefficient for the GSS in Serbian population were adequate for first 7 items. The CI-TCs were the lowest for items #8, #9 and #10 and they ranged from 0.29 to 0.38. Hotelling’s T-Squared test was statistically significant (HT^2^; F = 114.62; p<0.001).

### Construct validity

On EFA, Serbian version of the GSS showed 2 factors i.e. domains corresponding to the construct of the original scale (Table 4). Total variance explained by these two extracted factors was 48.92%Factor 1 (37.69% of total variance) contained 6 items, while Factor 2 (11.23% of total variance) was formed of 4 items. These two factors corresponded to the original SYMP-6 and FUNC-4 domains. Nevertheless, item distribution of the Serbian GSS did show some minor dissimilarity with the English version. The item #5 “Soreness, tiredness” from the original SYMP-6 scale was clustered in our FUNC-4 scale. On the contrary, item #8 “Hard to see in daylight” that belonged to the original FUNC-4 subscale was distributed in our SYMP-6 subscale. Communalities were higher than 0.40 for almost all items. Only the item #8 “Hard to see in daylight” had communality index lower than the adequate value of 0.4.

**Table 4:** Exploratory factor analysis of Serbian version of Glaucoma Symptom scale for both eyes with communalities.

GSS items	Factor 1 analogous to SYMP-6	Factor 2 analogous to FUNC-4	Communalities
1. Burning, Smarting, Stinging	**0.727**	0.282	0.608
2. Tearing	**0.742**	0.183	0.584
3. Dryness	**0.720**	0.184	0.552
4. Itching	**0.744**	-0.180	0.553
7. Feeling of Something in Your Eye	**0.626**	0.382	0.538
8. Hard to See in Daylight	**0.279**	0.264	0.147
5. Soreness, Tiredness	0.422	**0.500**	0.427
6. Blurry/Dim vision	0.252	**0.668**	0.510
9. Hard to See in Dark Places	0.003	**0.745**	0.555
10. Halos around lights	0.151	**0.628**	0.417

GSS—Glaucoma Symptom Scale

### Criterion validity

All subscale scores as well as GSS total score correlated positively (p<0.05) with the total NEI-VFQ 25 score (Table 5). Likewise, strong correlations were detected between analogous NEI-VFQ and GSS subscale scores. In particular, all GSS subscales and total score correlated with ocular pain, color vision, near and distance vision activities, vision specific mental health and role dependency (p = 0.001). Out of all NEI-VFQ 25 domains only vision specific dependency and driving were not correlated with GSS subscales and total score.

**Table 5:** Correlation of Glaucoma Symptom Scale subscales and total score for each eye and both eyes with NEI-VFQ 25.

Parameters		Symp6 Better Eye	Funct4 Better Eye	Total GSS BE	Sympt6 Worse Eye	Fuct4 Worse Eye	Total GSS WE	Sympt6 RE+LE	Funct4 RE+LE	Total GSS RE+LE
General health	ρ	0.119	0.080	0.109	0.184	0.147	0.181	0.158	0.106	0.154
	p	0.113	0.288	0.148	**0.014**	0.052	**0.016**	**0.036**	0.162	**0.044**
General vision	ρ	0.083	0.135	0.113	0.085	0.198	0.130	0.097	0.192	0.147
	p	0.273	0.073	0.135	0.258	**0.008**	0.084	0.198	**0.011**	0.050
Ocular pain	ρ	0.488	0.266	0.450	0.535	0.286	0.490	0.517	0.294	0.486
	p	**0.001**	**0.001**	**0.001**	**0.001**	**0.001**	**0.001**	**0.001**	**0.001**	**0.001**
Near vision	ρ	0.162	0.361	0.236	0.183	0.422	0.279	0.185	0.383	0.276
	p	**0.031**	**0.001**	**0.002**	**0.027**	**0.001**	**0.001**	**0.014**	**0.001**	**0.001**
Distant vision	ρ	0.162	0.361	0.236	0.183	0.422	0.279	0.185	0.383	0.276
	p	**0.031**	**0.001**	**0.002**	**0.027**	**0.001**	**0.001**	**0.014**	**0.001**	**0.001**
Social function	ρ	0.070	0.156	0.087	0.150	0.233	0.188	0.126	0.199	0.151
	p	0.354	**0.038**	0.252	**0.047**	**0.002**	**0.012**	0.096	**0.008**	**0.045**
Mental health	ρ	0.196	0.318	0.274	0.190	0.339	0.273	0.204	0.346	0.294
	p	**0.009**	**0.001**	**0.001**	**0.011**	**0.001**	**0.001**	**0.006**	**0.001**	**0.001**
Role limitation	ρ	0.186	0.267	0.251	0.217	0.337	0.294	0.226	0.296	0.293
	p	**0.013**	**0.001**	**0.001**	**0.004**	**0.001**	**0.001**	**0.003**	**0.001**	**0.001**
Depend. on others	ρ	0.051	0.020	0.023	0.084	0.084	0.086	0.067	0.068	0.052
	p	0.500	0.795	0.757	0.268	0.256	0.256	0.379	0.365	0.493
Driving difficulty	ρ	0.076	0.125	0.075	0.078	0.116	0.066	0.097	0.117	0.088
	p	0.312	0.096	0.320	0.303	0.124	0.382	0.200	0.122	0.243
Color vision	ρ	0.157	0.223	0.171	0.162	0.225	0.189	0.155	0.219	0.187
	p	**0.037**	**0.003**	**0.017**	**0.032**	**0.003**	**0.012**	**0.039**	**0.001**	**0.013**
Peripheral vision	ρ	0.141	0.195	0.182	0.080	0.225	0.130	0.123	0.214	0.179
	p	0.061	**0.009**	**0.015**	0.291	**0.003**	0.086	0.104	**0.004**	**0.017**
Total NEI VFQ	ρ	0.355	0.368	0.402	0.384	0.457	0.449	0.391	0.412	0.454
	p	**0.001**	**0.001**	**0.001**	**0.001**	**0.001**	**0.001**	**0.001**	**0.001**	**0.001**

ρ—Spearman's coefficient rho; p—probability level; BE–better eye; WE–worse eye; RE–right eye; LE–left eye; GSS—Glaucoma Symptom Scale; Depend–dependency; Bold–significant

The GSS total score and almost all subscale scores (better eye, worse eye, both eyes) negatively correlated (p<0.05) with better eye and worse eye MD, better eye sLV, better and worse eye glaucoma stage (Table 6). In contrast, very limited or no correlations were recorded between the GSS scores and other clinical parameters (number of glaucoma medications, number of drops per day and number of previous trabeculectomies).

**Table 6:** Correlation of Glaucoma Symptom Scale subscales and total score for each eye and both eyes with patients’ clinical characteristics.

Parameters		Symp6 BE	Funct4 BE	Total GSS BE	Sympt6 WE	Fuct4 WE	Total GSS WE	Sympt6 RE+LE	Funct4 RE+LE	Total GSS RE+LE
BE VA	ρ	-0.127	-0.132	-0.112	-0.172	-0.201	-0.156	-0.164	-0.170	-0.155
	p	0.093	0.079	0.138	**0.022**	**0.007**	**0.038**	**0.029**	**0.023**	**0.039**
WE VA	ρ	-0.093	-0.219	-0.148	-0.153	-0.253	-0.215	-0.137	-0.238	-0.191
	p	0.218	**0.003**	**0.049**	**0.042**	**0.001**	**0.004**	0.069	**0.001**	**0.011**
BE MD	ρ	-0.149	-0.194	-0.199	-0.109	-0.203	-0.169	-0.136	-0.222	-0.198
	p	**0.048**	**0.010**	**0.008**	0.150	**0.007**	**0.025**	0.070	**0.003**	**0.008**
WE MD	ρ	-0.083	-0.221	-0.159	-0.075	-0.210	-0.150	-0.088	-0.234	-0.167
	p	0.274	**0.003**	**0.035**	0.324	**0.005**	**0.047**	0.243	**0.002**	**0.026**
BE sLV	ρ	-0.157	-0.223	-0.211	-0.103	-0.198	-0.157	-0.139	-0.222	-0.194
	p	**0.038**	**0.003**	**0.005**	0.176	**0.009**	**0.038**	0.066	**0.003**	**0.010**
WE sLV	ρ	-0.098	-0.059	-0.094	-0.065	-0.076	-0.067	-0.088	-0.064	-0.081
	p	0.197	0.436	0.218	0.392	0.317	0.378	0.247	0.400	0.287
BE GL stadium	ρ	-0.151	-0.141	-0.179	-0.105	-0.175	-0.157	-0.135	-0.177	-0.178
	p	**0.045**	0.061	**0.017**	0.164	**0.020**	**0.037**	0.074	**0.018**	**0.018**
WE GL stadium	ρ	-0.098	-0.059	-0.094	-0.065	-0.076	-0.067	-0.088	-0.064	-0.081
	p	0.197	0.436	0.218	0.392	0.317	0.378	0.247	0.400	0.287
BE no medics	ρ	-0.087	-0.160	-0.137	-0.065	-0.177	-0.116	-0.086	-0.169	-0.135
	p	0.247	**0.034**	0.070	0.387	**0.019**	0.123	0.255	**0.024**	0.074
WE no medics	ρ	-0.042	-0.115	-0.079	-0.087	-0.179	-0.139	-0.079	-0.147	-0.123
	p	0.577	0.128	0.295	0.249	**0.017**	0.066	0.294	0.051	0.102
BE no drops	ρ	-0.080	-0.144	-0.116	-0.043	-0.170	-0.077	-0.074	-0.160	-0.170
	p	0.295	0.058	0.127	0.577	0.025	0.313	0.331	**0.035**	0.160
WE no drops	ρ	-0.019	-0.107	-0.061	-0.096	-0.189	-0.142	-0.076	-0.150	-0.117
	p	0.803	0.162	0.427	0.208	**0.012**	0.061	0.321	**0.048**	0.124
BE trab history	ρ	-0.017	-0.080	-0.029	-0.071	-0.071	-0.089	-0.047	-0.065	-0.059
	p	0.824	0.288	0.700	0.351	0.351	0.240	0.533	0.388	0.433
WE trab history	ρ	-0.042	-0.101	-0.055	-0.069	-0.143	-0.090	-0.091	-0.133	-0.111
	p	0.576	0.182	0.470	0.360	0.057	0.235	0.229	0.078	0.142

ρ—Spearman's coefficient rho; p—probability level; BE–better eye; WE–worse eye; RE–right eye; LE–left eye; GSS–Glaucoma Symptom Scale; medics–glaucoma medications; trab–trabeculectomy; GL–glaucoma; VA–visual acuity; MD–mean defect; sLV–square root of loss variance; Bold–significant

### Psychometric properties in Rasch analysis

Rasch analysis showed that the reliability index for GSS items is reasonably high indicating adequate reproducibility. Item separation index (3.04) shows that GSS can test up to three levels of difficulty i.e. severity of symptoms/complaints. This is good as GSS can consequently be used for glaucoma patients with different abilities and stages. On the other hand, GSS person separation index was 0.5 which was quite low (PSR 0.2), indicating that the questionnaire could not differentiate well participants’ abilities and their influence on quality of life (Table 7).

**Table 7:** Rasch analysis GSS fit statistics.

**Person** = 177	Total	Count	Measured		INFIT		OUTFIT	
			Measure	Realse	IMNSQ	ZSTD	OMNSQ	ZSTD
Mean	876.8	10.0	0.13	0.13	0.99	0.1	1.06	0.1
P.SD	135.6	0.1	0.09	0.17	0.44	1.0	0.72	1.1
Real RMSE	0.21	True SD	0.01	Separation	0.50	Person reliability		0.20
**Item** = 10	Total	Count	Measured		INFIT		OUTFIT	
			Measure	Realse	IMNSQ	ZSTD	OMNSQ	ZSTD
Mean	15520.0	177.0	0.1	0.1	1.03	0.2	1.06	0.1
P.SD	861.2	0.1	0.1	0.1	0.27	1.8	0.39	1.6
Real RMSE	0.01	True SD	0.01	Separation	3.04	Item reliability		0.90

P.SD—population standard deviation; REALSE—standard errors of measure estimates; Z—z standardized scores; STD—standard deviation; I—infit; O—outfit; MNSQ—mean square standardized residuals

According to a very low standard error of measurement (Model SE), we observed that the Serbian GSS showed appropriate reliability of items. Obtained correlation coefficients which were all very close to expected suggested adequate metric characteristics. Only one item (#8) had infit and outfit mean squares above the set referral range (0.5 to 1.5) (Table 8).

**Table 8:** Rasch analysis GSS item statistics.

Entry number	Total score	Total count	Measure	Model SE	INFIT		OUTFIT		PTMEASURE	
					MNSQ	ZSTD	MNSQ	ZSTD	Correl.	Expect.
1	15275	177	0.01	0.01	0.73	2.6	0.65	2.4	0.53	0.49
2	15300	177	0.01	0.01	0.86	1.2	0.87	0.7	0.51	0.49
3	13575	177	0.03	0.01	0.76	2.4	0.76	2.3	0.67	0.64
4	14975	177	0.01	0.01	0.98	0.1	1.02	0.2	0.53	0.52
5	16050	177	0.01	0.01	1.01	0.1	0.88	0.5	0.41	0.42
6	15925	177	0.01	0.01	0.94	0.4	1.15	0.7	0.43	0.43
7	15350	177	0.01	0.01	0.84	0.9	0.84	0.9	0.51	0.49
8	16750	177	0.02	0.01	1.61	2.6	2.04	2.4	0.25	0.33
9	15375	177	0.01	0.01	1.44	3.4	1.46	2.4	0.43	0.48
10	16625	177	0.02	0.01	1.07	0.4	0.92	0.1	0.34	0.35
Mean	15520	177	0.01	0.01	1.03	0.2	1.06	0.1	/	/
P.SD	861.2	177	0.01	0.01	0.27	1.8	0.39	1.6	/	/

P.SD—population standard deviation; SE—standard error; MNSQ—mean square standardized residuals; Z—z standardized scores; STD—standard deviation; Correl—correlation; Expect—expected.

**Fig 1:**
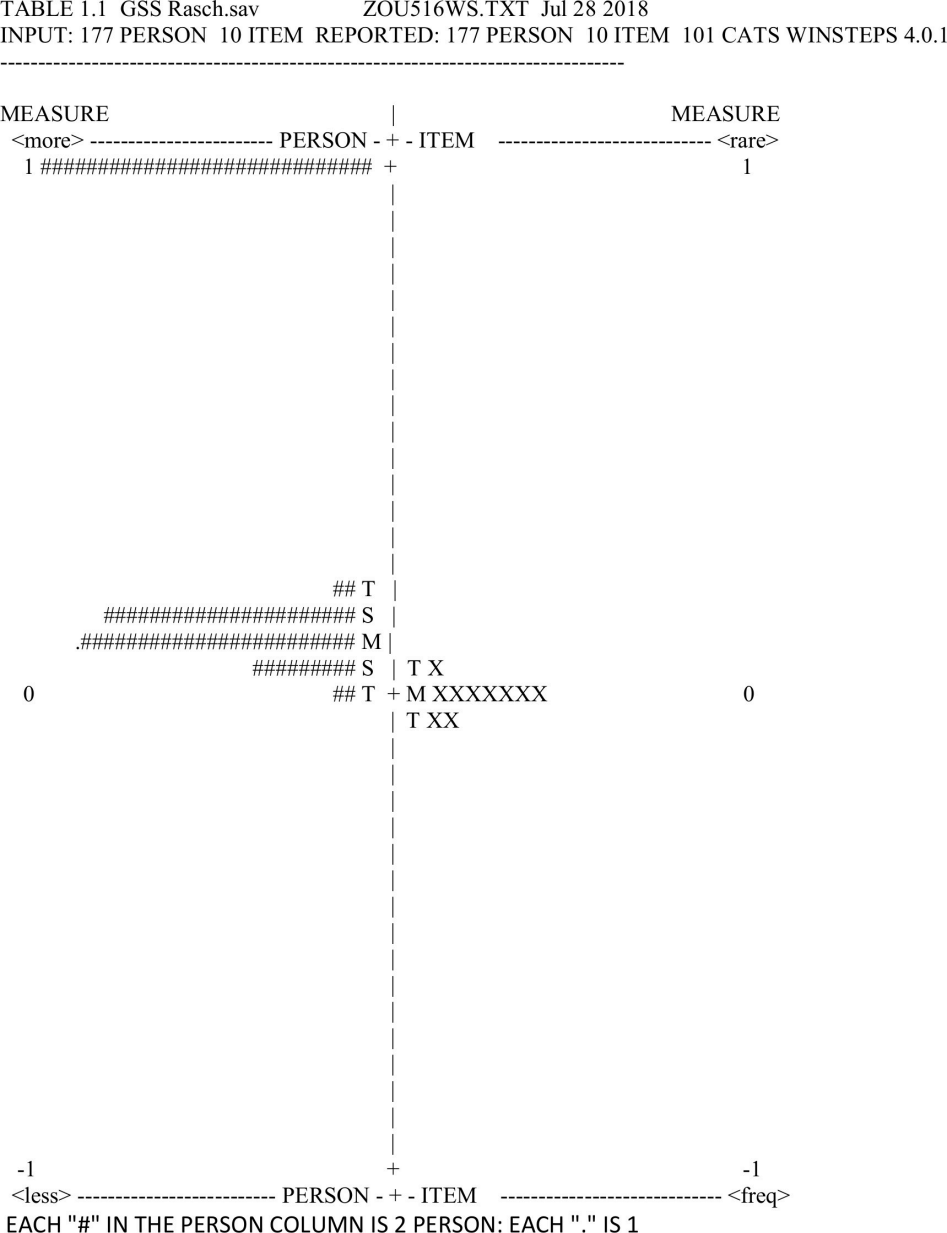
GSS Rasch analysis person item chart.

In Fig 1 we observed that mean of the item difficulty and patient ability were around the same level, close to 0 logit and the difference between the two means was not higher than 1 logit. This indicates that item difficulties generally corresponded to the abilities of the studied patients. However, Fig 1 also reveals that a distinct group of patients were positioned high in the scale (so-called ceiling effect) meaning that they did not report major disability. This ceiling effect indicated that GSS had poor targeting for this group of patients.

Moreover, chart shows that seven GSS items had the same mean level of difficulty/intensity, while one symptom (#3 “Dryness”) was more and two symptoms (#8 “Hard to See in Daylight” and #10 “Halos around light”) less prominent/important for investigated glaucoma patients. Symptoms #8 and #10 seem to occur more often in patients with less ability potentially due to more advances glaucoma stage. Contrary, dryness of the eyes was the symptom on which all tested glaucoma patients complained. Therefore, it might be the symptom to appear even in the early glaucoma stage, probably due to the effect of glaucoma medications.

## Discussion

The total scores for both eyes and each eye separately were higher in Serbian population than the average values in other reports [17, 18]. Unlike in the original study, SYMP-6 subscale had lower mean values than FUNC-4 and, interestingly, this finding was also noted in Italian population [18].

As for the GSS psychometric properties our study is mostly in accordance with the previous studies that used both CTT and Rasch analysis. Based on the observed findings in this study, both statistical approaches revealed some advantages and some drawbacks of the GSS. Nevertheless, Rasch analysis, in comparisons with CTT, suggested that the GSS had suboptimal psychometric characteristics.

In line with the presented results, assessment using the CTT indicates that Serbian version of the GSS has overall desirable metric characteristics. Cronbach’s alpha coefficient for the total GSS in Serbian language was proper (>0.80) and thus, internal consistency of Serbian GSS can be labeled appropriate. The Cronbach’s alpha coefficient observed in our study was consistent with coefficients in the original scale [17], but also in other populations [18, 19]. When GSS domains were analyzed, Cronbach’s alpha coefficients for SYMP-6 subscale were also greater than 0.8, meaning that items in this domain comprise one meaningful and consistent construct related to non-visual symptoms. However, FUNC-4 subscale had low internal consistency with a Cronbach’s alpha coefficient of 0.53.

Consistent with the original scale [17], Serbian version of the GSS also showed 2 domains on EFA. This means that the GSS clearly separates two distinctive underlying constructs. Additionally, stability of the scale suggested that answers obtained at initial testing corresponded to answers reported at re-testing. These findings indicate that perception of glaucoma-related symptoms and functions remains similar over time. According to Rasch analysis, we observed that GSS reliability index for items was quite high, suggesting adequate reproducibility. Item fit statistics were satisfactory, with almost all items corresponding to Rasch model expectations, indicating reasonable validity. When person-item scale was evaluated, mean item difficulty corresponded to mean person ability, which is a characteristic of a proper targeting. Rash analysis showed that for Serbian glaucoma patients three groups of items could potentially be formed in terms of symptoms difficulty/intensity which is similar to glaucoma stages once again showing adequate construct of the GSS.

Criterion validity of the Serbian version of GSS was tested by correlating total GSS and subscale scores with domains of the NEI-VFQ 25 questionnaire. Good association was found between domains of the GSS and analogous domains of the NEI-VFQ 25 [35]. No correlations were found between the subscales that capture dissimilar constructs (e.g. SYMP-6 and FUNC-4 with NEI-VFQ 25 General health, Vision specific dependency and Driving). Our results are in accordance with the previous findings from the original and Italian validations [17, 18].

Contrary to previous two validation studies [18, 20] our version of the GSS corresponded to measures of disease severity. Strong correlations were observed between better, worse eye and both eyes GSS outcomes with glaucoma parameters (better and worse eye MD, better eye sLV and staging). However, correlation was not statistically significant when worse eye sLV was compared with the questionnaire total and subscale scores. This was not surprising because SLV can be paradoxically low in patients with advanced glaucoma [44]. Moreover, occurrence of some symptoms is not related to disease stage (present all the time). Interestingly, similar to the original study [17] and in Italian validation [18] minimal or no correlations were registered between the GSS and the glaucoma treatment variables (number of glaucoma medications/drops, trabeculectomy history). Literature evidence previously showed that the presence of OSD symptoms do not correlate with the severity of OSD changes [45].

On the other hand, although overall GSS appears adequate, we observed some distinct validity issues indicated by the CTT and later confirmed by Rasch analysis.

Rasch analysis showed that the GSS was not able to differentiate person abilities (low PSR and PSI) suggesting that the GSS had low discriminative ability. This result of poor measurement precision was also reported in two previous Rasch validations in Indian and Australian populations [20,21].

The effect of glaucoma stage strongly affected the GSS validity and consequently might cause a problem in terms of its clinical applicability. Rasch analysis revealed evident ceiling effect, with numerous patients being clustered in the upper half of the graph, suggesting ineffective targeting of the GSS for all glaucoma patients. These findings could suggest that the GSS was more sensitive in description of HRQoL of patients with more severe symptoms.

In fact, problematic features of the GSS could be in close relationship with the characteristics of tested patients and the GSS construct for wide glaucoma patient populations. Our sample comprised 42% of patients with early disease stage who were appropriately treated and consequently had fewer symptoms and better functional abilities. Using the GSS in mild to moderate glaucoma stages, who accounted for the largest proportion of participants in our sample, could explain somewhat suboptimal results observed in this study. Therefore, it seemed that GSS items were relatively easy i.e. not as impaired in our population of glaucoma patients and inadequate for testing deterioration of patient functioning in early stage glaucoma. This claim is substantiated by the finding that the examined patients mostly had better HRQoL and less visual and non-visual related complaints. Interestingly, similar finding of poor GSS properties when tested in population with early glaucoma and preserved vision was noted in Australian study [20]. Also, these aspects of HRQoL were exactly the ones tested by the FUNC dimension of the GSS. This might be the reason why on traditional assessment FUNC-4 subscale had low Cronbach’s alpha coefficient.

Furthermore, particular problem was observed with the item #8 “Hard to see in daylight” pertaining to the FUNC dimension. Items #8, #9 and #10 from FUNC factor also had low value of the CI–TC coefficient. Items #5 and #8 in our analysis changed their factor loading. Moreover, only item #8 did not have statistics that fit in the acceptable range as well as lower than adequate communality index. Still, even when item #8 was removed from the scale, Cronbach’s alpha coefficients for total scale and FUNC-4 subcale did not change significantly (0.81 and 0.54, respectively). This might also be the reason why the GSS construct was not observed as adequate on Rasch analysis. Moreover, it has to be noted that poor targeting is generally a problem of ophthalmology questionnaires since the goal of treatment is to minimize the symptoms of moderately impaired patients and ceiling effect on symptom measurement may be inevitable [40, 46, 47].

Consequently, based on these findings, the oddity of item #8 can potentially be attributed to particularities of our sample of Serbian glaucoma patients. In Serbian population it seems that item #5 “tiredness” was be perceived as impediment to daily functioning due to fatigue while item #8 “visual difficulties in daylight” as discomfort associated with blinding glare. The actual specificities of Serbian glaucoma patients and their differences in item comprehension could be explored in detail by further qualitative studies.

Nevertheless, based on the clinical work with glaucoma patients, daylight vision (item #8) was considered important because one of the most frequent complaints that our patients reported were difficulties related to lighting and, in particular, adaptation to different levels of light. Moreover, almost all glaucoma specific questionnaires include items that relate to glare disability or troubles connected with outdoor glare and indoor dark adaptation. Since the question that relates to dim light is already included in the GSS, this question is equivalent to difficulties in bright light. Nelson et al. [48] reported that best functional tests that correlate with perceived disability are glare disability. An analysis of patients enrolled in Collaborative Initial Glaucoma Treatment Study (CIGTS) [49] showed that over 50% of patients reported at least “some” difficulties in tasks involving glare. As a result, it was decided to keep the item #8 in this first translation and validation of the GSS for Serbian population. Moreover, in order to easily and adequately compare different populations, the same structure of questionnaire should be applied. Developing a slightly different version of the same instrument for every population and in every language is confusing for researchers who want to use a particular instrument with an established questionnaire title and modifications in each population based on the obtained results may limit direct comparison between studies in different countries and populations [16].

Thus far, a comprehensive, precise and valid glaucoma specific instrument has not yet been available as none of the original glaucoma specific questionnaires had good performances on Rasch analysis [28]. Still, our results indicate that the optimal instrument should entail more diverse items to represent all glaucoma patients’ condition. In order to improve this aspect of the GSS, inclusion of items that are more difficult i.e require more demanding tasks, or measure more specific symptoms or complaints is suggested. Currently, researchers/clinicians can overcome this issue by application of more questionnaires simultaneously with GSS when assessing health status, complaints and quality of life of glaucoma patients in Serbia. Further studies on larger samples that could be stratified according to glaucoma stage are needed to investigate more thoroughly.

In conclusion, although when analyzed with CTT Serbian version of the GSS seems to be a valid instrument for glaucoma patients, however serious measurement flaws of the GSS were observed when the IRT model was applied. Although almost all items had infit and outfit statistics in the referral range, suboptimal measurement precision and ceiling effect in targeting were registered. This finding suggests that application of the scale might reflect better symptoms and difficulties in patients who have later stages of glaucoma i.e. more intense symptoms. Similarly, because of suboptimal infit statistics of vision in daylight, it is recommended to consider potential modification of this particular item. This could be achieved based on clinical work with patients who could describe in more detail the difficulties that they encounter in daylight activities. Therefore, further studies are warranted prior to GSS application in clinical practice among Serbian glaucoma patients. Additional research could be used to potentially modify and improve the GSS to better suit Serbian glaucoma patients.

## Supporting information

S1 File Serbian glaucoma symptom scale. (PDF)
