# Supplementary material for: Glaucoma Symptom Scale: Psychometric properties of the Serbian version
Source: PLoS One. 2019 May 20;14(5):e0216920. doi: 10.1371/journal.pone.0216920 (PMC6527221; doi:10.1371/journal.pone.0216920)
Supplement: S1 File — (PDF) [file pone.0216920.s001.pdf]

Upitnik Skala simptoma kod glaukoma (GSS)

Da li ste u poslednje 4 nedelje imali neki od sledećih problema ?
(Molimo da odgovorite odvojeno za desno i za levo oko)

Problem	**Da**<br>**Koliko je bio neprijatan ?**				**Ne**
	**Veoma neprijatan**<br><br>**OD : OS**	**Određena neprijatnost**<br><br>**OD : OS**	**Vrlo mala neprijatnost**<br><br>**OD : OS**	**Nimalo neprijatan**<br><br>**OD : OS**	**OD :OS**
Pečenje,žiganje,probadanje					
Suzenje					
Suvoću					
Svrab					
Bol, zamor					
Zamagljen/Mutan vid					
Osećaj stranog tela u oku					
Teškoću da vidite na dnevnom svetlu					
Teškoću da vidite na tamnim mestima					
Oreole oko svetla					
